# The European Variation Archive: a FAIR resource of genomic variation for all species

**DOI:** 10.1093/nar/gkab960

**Published:** 2021-10-28

**Authors:** Timothe Cezard, Fiona Cunningham, Sarah E Hunt, Baron Koylass, Nitin Kumar, Gary Saunders, April Shen, Andres F Silva, Kirill Tsukanov, Sundararaman Venkataraman, Paul Flicek, Helen Parkinson, Thomas M Keane

**Affiliations:** European Molecular Biology Laboratory, European Bioinformatics Institute, Wellcome Genome Campus, Hinxton, Cambridge, UK; European Molecular Biology Laboratory, European Bioinformatics Institute, Wellcome Genome Campus, Hinxton, Cambridge, UK; European Molecular Biology Laboratory, European Bioinformatics Institute, Wellcome Genome Campus, Hinxton, Cambridge, UK; European Molecular Biology Laboratory, European Bioinformatics Institute, Wellcome Genome Campus, Hinxton, Cambridge, UK; European Molecular Biology Laboratory, European Bioinformatics Institute, Wellcome Genome Campus, Hinxton, Cambridge, UK; ELIXIR Hub, Wellcome Genome Campus, Hinxton, Cambridge, UK; European Molecular Biology Laboratory, European Bioinformatics Institute, Wellcome Genome Campus, Hinxton, Cambridge, UK; European Molecular Biology Laboratory, European Bioinformatics Institute, Wellcome Genome Campus, Hinxton, Cambridge, UK; European Molecular Biology Laboratory, European Bioinformatics Institute, Wellcome Genome Campus, Hinxton, Cambridge, UK; European Molecular Biology Laboratory, European Bioinformatics Institute, Wellcome Genome Campus, Hinxton, Cambridge, UK; European Molecular Biology Laboratory, European Bioinformatics Institute, Wellcome Genome Campus, Hinxton, Cambridge, UK; European Molecular Biology Laboratory, European Bioinformatics Institute, Wellcome Genome Campus, Hinxton, Cambridge, UK; European Molecular Biology Laboratory, European Bioinformatics Institute, Wellcome Genome Campus, Hinxton, Cambridge, UK

## Abstract

The European Variation Archive (EVA; https://www.ebi.ac.uk/eva/) is a resource for sharing all types of genetic variation data (SNPs, indels, and structural variants) for all species. The EVA was created in 2014 to provide FAIR access to genetic variation data and has since grown to be a primary resource for genomic variants hosting >3 billion records. The EVA and dbSNP have established a compatible global system to assign unique identifiers to all submitted genetic variants. The EVA is active within the Global Alliance of Genomics and Health (GA4GH), maintaining, contributing and implementing standards such as VCF, Refget and Variant Representation Specification (VRS). In this article, we describe the submission and permanent accessioning services along with the different ways the data can be retrieved by the scientific community.

## INTRODUCTION

Understanding how genetic variation leads to phenotypic differences is the cornerstone of genome biology. Genetic variants span individual base changes, insertions-deletions of a few bases, structural variations of tens or hundreds of kilobases (e.g. inversions, insertions, deletions, translocations), and up to whole chromosome karyotype differences. The advances in genome sequencing have resulted in an important increase in the number of studies of genetic variants applied to the evolution of species, the betterment of human health, and the improvement of food security. To make full use of these datasets, they need to be available and indexed in a public database.

The European Variation Archive (EVA) is a primary open repository for archiving, accessioning and distributing genetic variation, including single nucleotide variants (SNVs), short insertions and deletions (indels), and larger structural variants (SVs) in any species. Services to researchers include permanent archiving of variants; calculation of variant annotation; and an intuitive browser and REST API to view and query variants from a specific research experiment or across an entire species. The EVA, the NCBI-based dbSNP (1) and dbVar (2) databases, and the Chinese Genome Variation Map resource (3) form a worldwide network for brokering submissions, assigning permanent study and locus identifiers and exchanging these identifiers to ensure that consistent data is available at all sites. In late 2016, dbSNP and dbVar announced that they would cease accepting non-human submissions and since then the EVA maintains variant locus accessions (rs IDs) for all non-human species. Reference SNP IDs, or rs IDs, are unique identifiers that are assigned to a group of submitted variants that co-locate to the same position and variant type on a species’ genome and therefore are fundamental for cross referencing and findability of genetic variants.

The EVA is a FAIR (Findable, Accessible, Interoperable, Reusable) (4) data sharing resource whose core goal is to make genetic variation data as widely accessible and interoperable as possible to maximise reuse by the scientific community. The EVA actively contributes and implements international standards for variant exchange, modelling, and annotation. The EVA is one of the inaugural Driver Projects in the Global Alliance for Genomics and Health (GA4GH), either actively maintaining genomic data standards (e.g. Variant Call Format - VCF) or contributing to their development (e.g. Refget (5), VRS (6)) to improve interoperability for genetic variation data. The EVA is a recognised ELIXIR Deposition Database, which provides guidance to those who formulate policy and working practices about the appropriate repositories for publishing open data ensuring that published data is permanently findable. The EVA contributes to the ELIXIR Plant Sciences and ELIXIR Human CNV communities to improve metadata submission standards and variant exchange for these use-cases.

## EVA RESOURCE

**Figure 1.**
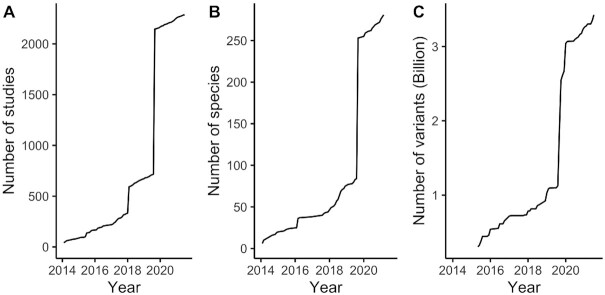
Growth of EVA since 2014. (**A**) Number of studies and (**B**) new species. (**C**) Growth of EVA in variants (variant ingestion only started in 2015).

The EVA was established at EMBL-EBI in 2014, incorporating and extending the SV-only Database of Genomic Variants archive (DGVa). Advances in genome technologies largely removed practical distinctions between larger and smaller types of genome variation which created a pressing need for a single archive encompassing all types of variants. The EVA now contains 2,287 studies and 3.4 billion variants across 281 species (Figure 1A–C). A notable step change in the size of the archive happened in 2019 when EVA imported all non-human genetic variants and studies from dbSNP. Almost half (47%) the studies deposited in EVA are relatively small with less than 1000 variants, 31% have between 1000 and 1M variants and 21% have more than 1M. The EVA’s species diversity continues to grow with plant and mammals driving most of the recent increase (Supplementary Figure S1).

### Data submission

**Figure 2.**
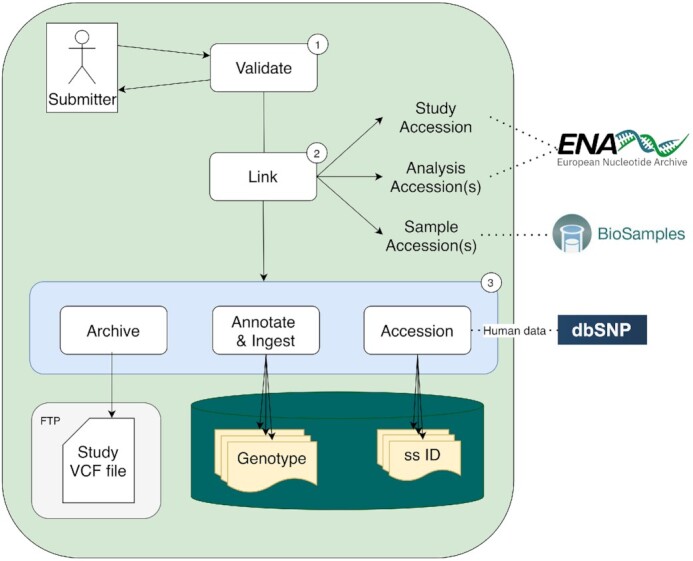
Description of the submission process for a single study: (1) Validation is undertaken with the submitter to ensure consistency with EVA’s standards. (2) The submission is linked to other EMBL-EBI resources. (3) The ingestion process archives, accessions and annotates the variants. Special case is made for human data for which the submitted variant accessioning is delegated to dbSNP.

Ensuring data consistency upon submission is an essential step to ensure interoperability and support cross-study comparative genomics. Before accepting a submission, the EVA carries out validation steps for data and metadata, for example verifying that the submitted information adheres to the VCF specification (7), the variants are mapped to a reference sequence registered by the International Nucleotide Sequence Database Collaboration (INSDC) (8), the reference base in the VCF record matches the reference sequence and the study metadata is consistent with the SRA data model (https://github.com/enasequence/schema). Additionally, submitted variants are required to be supported by either experimentally determined sample genotypes or population allele frequencies. The EVA then creates links for each submission to associate the metadata with other resources such as BioSamples (9) for phenotype metadata and the European Nucleotide Archive (10) for the raw data (Figure 2). These requirements ensure the submission follows standards used by the community, enhancing the FAIR-ness of EVA data.

The ingestion process (Figure 2) is composed of three steps. First, the validated VCF file is archived and made available immediately. Second, a unique SubSNP identifier (ss ID) is assigned to each variant reported in a particular study (Figure 2); the same variant reported across multiple studies receives a unique ss ID for each new study. Finally, each variant and its genotype are annotated using the Ensembl Variant Effect Predictor (11) and stored individually in the EVA’s databases. This allows the EVA to provide the original submission faithfully to build a queryable database of variants and genotypes across all the submissions. In the case of a submission containing human data, the submitted VCF file is not accessioned by EVA but is brokered to dbSNP that will issue both ss IDs and rs IDs.

### Data access

**Figure 3.**
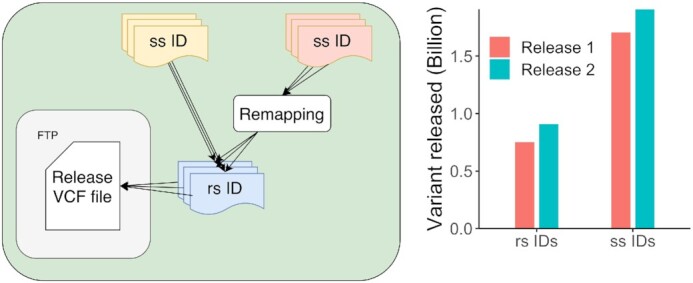
(left) Diagram describing the clustering of submitted variants from multiple studies with optional remapping to ensure that all variants are on the same reference. (right) Number of rs IDs and ss IDs exported during the EVA first two releases.

Since 1998, the rs scheme has been used to cross-reference genetic variants between genomics resources. The submitted variants and their ss IDs are remapped between genome builds per species to ensure that they are described relative to the latest reference so that newly and existing variants can be compared (full details on the EVA remapping pipeline: https://github.com/EBIvariation/variant-remapping). Remapped submitted variants are periodically clustered and assigned locus identifiers (rs IDs) (Figure 3). The resulting clustered variants are exported into a release VCF including study accessions for all ss IDs (Figure 2). In its second release the EVA assigned more than 938M locus accessions across 218 species (Figure 3). From its 3rd release the EVA will remap all submitted variants to newer assemblies (synchronised with the Ensembl browser for overlapping species).

For bulk access to the archived data, the EVA also provides VCF files as they were submitted and validated alongside the ss IDs that were assigned (Figure 2). The EVA’s web services usage has been growing steadily since its inception to reach 3M requests from 10K unique IPs in 2020 (Supplementary Figure S2). Our daily usage is routinely 1500 requests emanating from 60 unique IPs (Supplementary Figure S3). The FTP which hosts the archived studies and the EVA releases has also seen an important increase and in 2020 the release data was accessed 44K times from 531 unique locations (Supplementary Figure S2).

All data archived in EVA is also available for exploration on the EVA website (https://www.ebi.ac.uk/eva/) via the study and variant browsers. The study browser provides a high level overview of the studies metadata and publication links enabling users to find studies for a given species or class of variants. The variant browser allows the user to search for variants by position, gene or variant accessions (rs ID or ss ID) and provides details about the predicted consequences or population frequencies in each study the variant is found in. A REST API is available to support programmatic access to all the study metadata, variants and genotypes to encourage integration with genome browsers and other species-specific portals. Two of these endpoints implement GA4GH standardised protocols for discovery and access: Beacon (12) provides a mechanism to determine the presence or absence of a particular allele in a dataset and htsget (13) enables streaming of variant data from genomic regions of interest.

## DISCUSSION

The EVA is committed to implementing the FAIR principles for genetic variation data by providing long term access to each submitted study, sample, and variant. Each sample is registered with the BioSamples database which encourages cross references with other resources (e.g. multi-omics experiments). The EVA uses open standards to disseminate genetic variant data and actively contributes to their improvements. We have been active contributors to several GA4GH standards and co-maintainer of the VCF format specification, and provide a VCF format validator (https://github.com/EBIvariation/vcf-validator/) for EVA submitters. Known issues with this format include having alternate representations of the same variant (e.g. indels), complex multi-allelic variants, and structural variation representation. These issues particularly affect EVA since we receive submissions from a wide array of submitters. If not accounted for, this can potentially have a detrimental effect on multi-sample comparative analysis (e.g. allele frequencies). Community conventions to this problem include normalising and left-aligning indels prior to comparative analysis, splitting multi-allelic variants into constituent simple variants, and recent proposals to improve SV representation in VCF 4.4. The dbSNP SPDI and GA4GH VRS address the issue of ambiguous variant representation using the Variant Overprecision Correction Algorithm (VOCA) which provides additional reference context bases to remove ambiguity.

The widespread use of third generation long read sequencing technologies means that it is now possible to generate reference quality genomes for almost any species. Initiatives such as the Earth Biogenome Project (14), Genome 10K Project (15) and Bat 1K (16) will dramatically increase the number of species with high quality genomes across all domains of life. This will enable researchers to use short read sequencing to sample genetic variations and study population genetics. It is essential that the underlying genetic variants that support population studies are publicly accessible to support reproducibility and data reuse. Accordingly, we expect that demand for accessioning and sharing genetic variants will increase dramatically in coming years and see the EVA playing a critical role to support both scientists and data aggregation resources (e.g. genome browsers, species-specific portals). A key challenge for the EVA will be to ensure that our underlying technology and processes scale to continue to meet the increased demand.

In the past 10 years, draft reference genomes have been produced from second generation sequencing technologies for many species, e.g. key agricultural species such as pig (17), and model organism strains (18). Many of these genomes are now being upgraded to be reference quality using long reads, and these assemblies are orders of magnitude more contiguous and drastically reduce the proportion of unknown bases (19). While the EVA RS release process maps previously identified genetic variants onto these new reference genomes, in cases where there are very significant changes (e.g. many new haplotypes) to the underlying reference genome it is more appropriate to re-align raw reads and re-call SNPs and indels (19). Similarly, as genome annotation is updated for existing or new reference genomes, the EVA periodically updates our Variant Browser with new functional consequences via the Ensembl Variant Effect Predictor (11).

The concept of a single reference genome per species has been a core principle in comparative genome analysis. However the availability of multiple reference quality genomes per species and population scale collections of genetic variants (e.g. UK Biobank) is challenging this assumption. We are seeing the emergence of graph-based genome representations to efficiently store and represent collections of variants and genomes of related individuals or strains (20). A key feature of these is that they enable discovery and representations of genetic variation on non-reference haplotypes. Projects such as the Human Pangenome Reference Consortium (HPRC) (21) are making great advances in methods for detecting, storing, representing, and cross referencing variants in genome graphs. The EVA will continue to engage with relevant groups (e.g. Genome browsers, GA4GH VRS) towards community standards for how we will represent and share graph based genetic variants.

## DATA AVAILABILITY

The European Variation Archive can be accessed via: https://www.ebi.ac.uk/eva/. Content is distributed under the EMBL-EBI Terms of Use available at (https://www.ebi.ac.uk/about/terms-of-use).

## Supplementary Material

gkab960_Supplemental_File
