# Supplementary material for: The European Variation Archive: a FAIR resource of genomic variation for all species
Source: Nucleic Acids Res. 2021 Oct 28;50(D1):D1216–20. doi: 10.1093/nar/gkab960 (PMC8728205; doi:10.1093/nar/gkab960)
Supplement: gkab960_Supplemental_File [file gkab960_supplemental_file.pdf]

Species diversity in EVA datasets **1**

EVA data access statistics **2**

Species diversity in EVA datasets

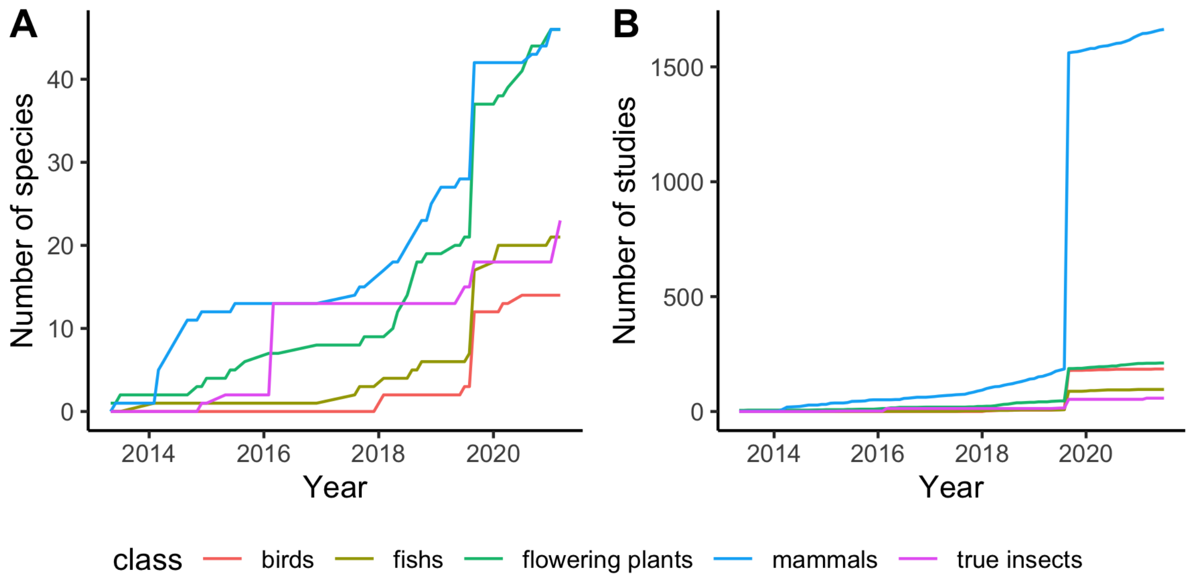

Supplementary figure 1: progression of the number of (A) species and (B) studies in the 5 most frequent class of Eukaryotes present in the EVA's data

Bacteria accounts for 38% of species diversity held in the EVA but they were almost exclusively imported from dbSNP and EVA received very few additional submissions. In the Eukaryotes, the EVA's data is dominated by mammals and flowering plants (supplementary figure 1) and mammals also account for the largest growth in number of studies. These trends are driven by commercial interest in agricultural species. As biodiversity and pathogen related studies become more important and affordable, we expect the EVA's datasets to be more diverse in the future.

EVA data access statistics

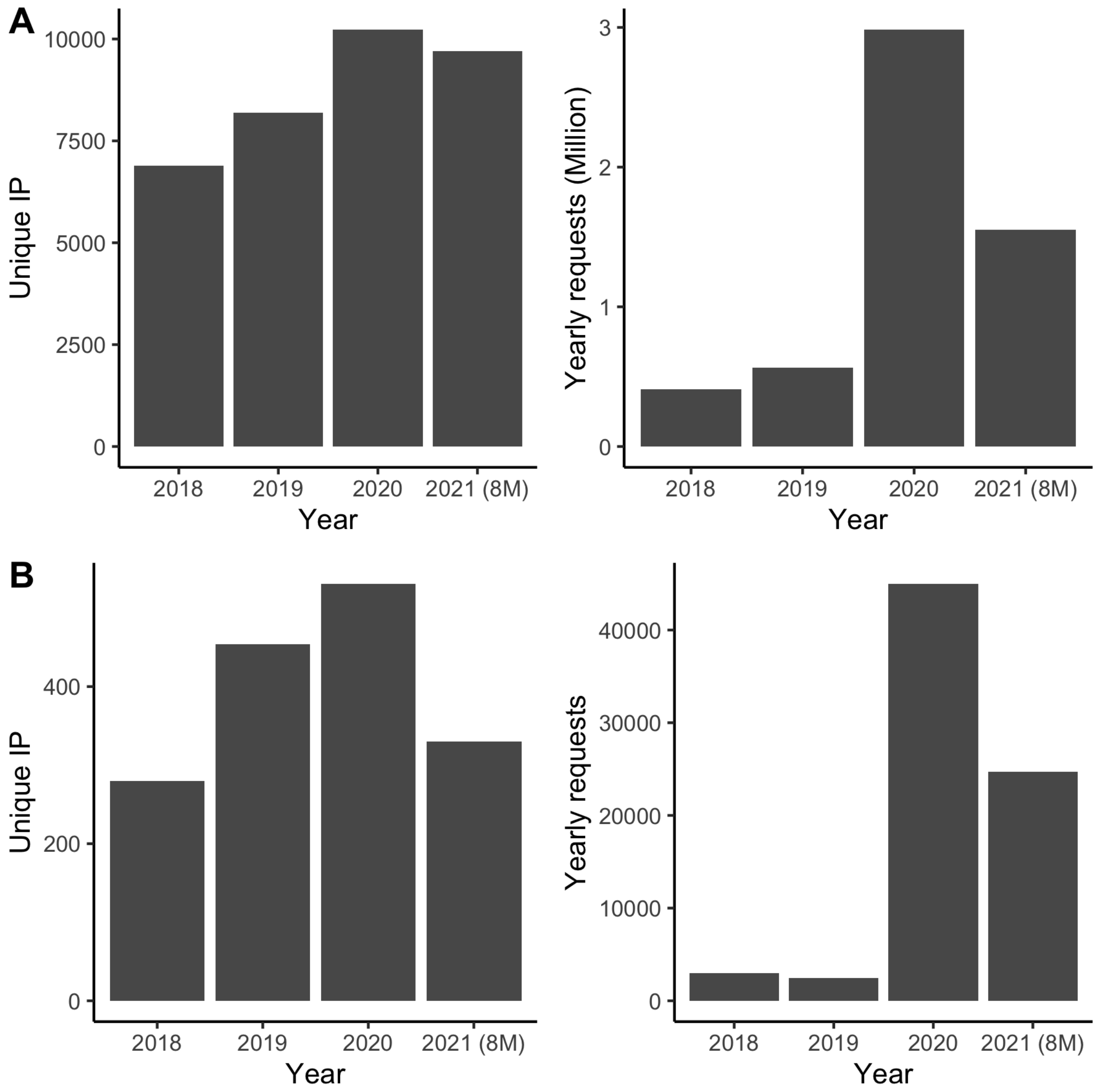

Supplementary figure 2: Unique IP connected (left), non unique connection (right) to the EVA web-services (**A**) and FTP (**B**) on a daily basis. 2021 only has data for the first 8 months of the year.

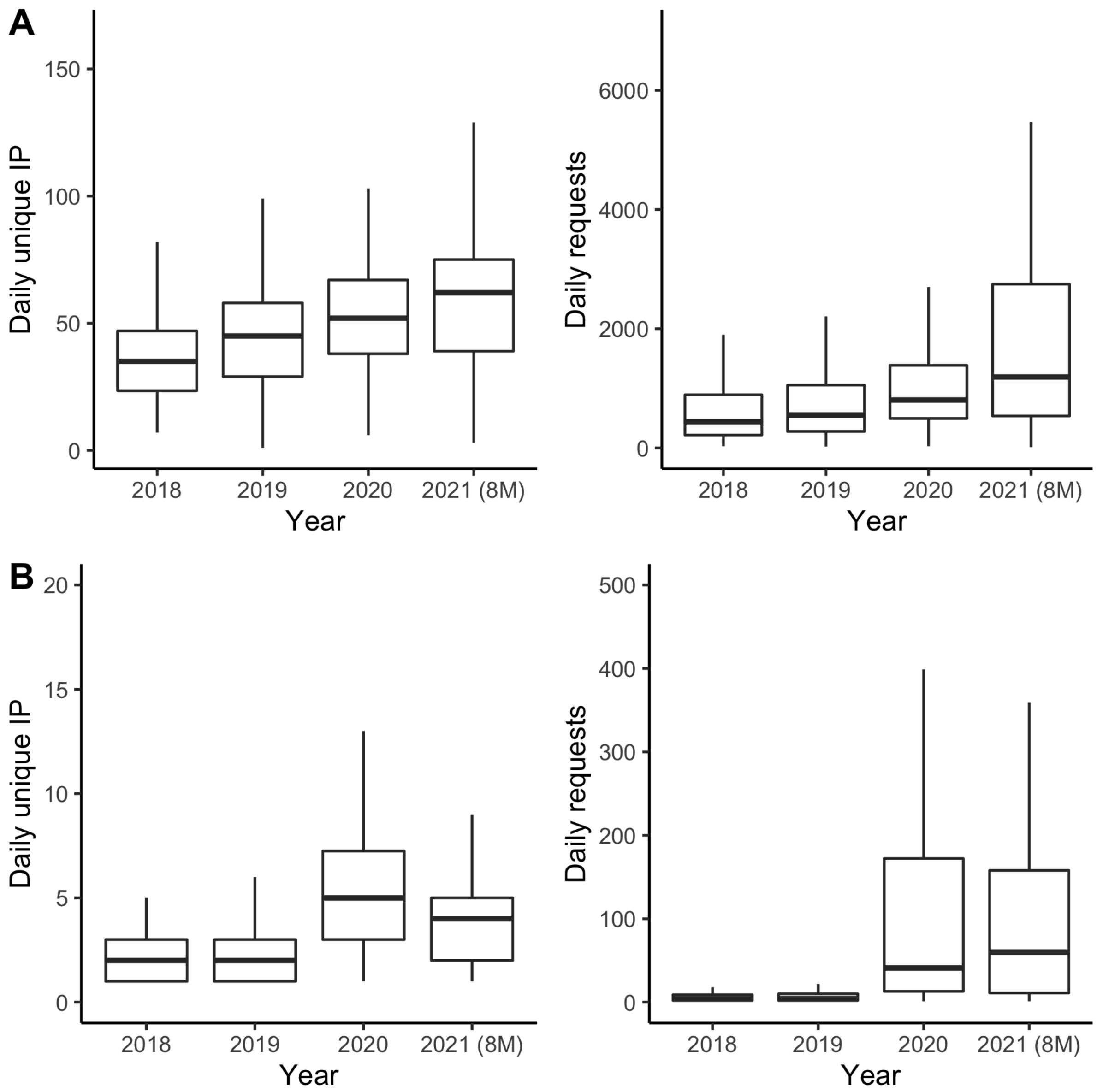

Supplementary figure 3: Unique IP connected (left), non unique connection (right) to the EVA web-services (**A**) and FTP (**B**) on a daily basis. Each box represents the distribution of each day with at least one connection. 2021 only has data for the first 8 months of the year.
